# The mutational landscape of quinolone resistance in *Escherichia coli*

**DOI:** 10.1371/journal.pone.0224650

**Published:** 2019-11-05

**Authors:** Kamya Bhatnagar, Alex Wong

**Affiliations:** Department of Biology, Carleton University, Ottawa, ON, Canada; Nitte University, INDIA

## Abstract

The evolution of antibiotic resistance is influenced by a variety of factors, including the availability of resistance mutations, and the pleiotropic effects of such mutations. Here, we isolate and characterize chromosomal quinolone resistance mutations in *E*. *coli*, in order to gain a systematic understanding of the rate and consequences of resistance to this important class of drugs. We isolated over fifty spontaneous resistance mutants on nalidixic acid, ciprofloxacin, and levofloxacin. This set of mutants includes known resistance mutations in *gyrA*, *gyrB*, and *marR*, as well as two novel *gyrB* mutations. We find that, for most mutations, resistance tends to be higher to nalidixic acid than relative to the other two drugs. Resistance mutations had deleterious impacts on one or more growth parameters, suggesting that quinolone resistance mutations are generally costly. Our findings suggest that the prevalence of specific *gyrA* alleles amongst clinical isolates are driven by high levels of resistance, at no more cost than other resistance alleles.

## Introduction

The increasing prevalence of antimicrobial resistance (AMR) has become an urgent public health problem worldwide. For example, resistance to ciprofloxacin, the most commonly purchased antimicrobial by hospitals in Canada between 2008–2014 [1], in *Escherichia coli* rose to 26.7% in 2015 from 21.6% in 2009 [2]. The present AMR crisis has been attributed to the misuse and overuse of antibiotics, as well as the scarcity of novel drug development [3–7]. Given the rapid increase in the prevalence of resistance, an understanding of the principles underlying resistance evolution is vital.

Adaptation, of which the evolution of AMR is a prime example, is driven by the interplay between mutation, selection, and demographic processes like drift. Mutation determines the rate at which beneficial variants are introduced into a population, while selection and demography govern the fates of these variants. Thus, in understanding the evolution of AMR, we are interested in both mutation and selection. We expect, for example, that higher mutation rates will generally lead to a more rapid evolution of resistance. The spread of a given mutation will then be influenced by its selective consequences, including its effect on resistance, and on its pleiotropic effects, such as fitness in the absence of antibiotic, and collateral sensitivity or cross resistance to other antibiotics.

The rate of mutation to resistance is given by the product of population size (*N*), overall mutation rate (*μ*), and the fraction (*f*) of mutations that are beneficial (i.e., those that grant resistance). Thus, resistance mutations will appear more frequently for larger populations, or for populations with higher mutation rates. Up to a point, this dependence on *Nμ* leads to an increase in the rate of adaptation; however, as *Nμ* approaches 1, a population is no longer limited by mutational input, but instead by competition between competing mutations (e.g., [8,9]).

Relevantly here, the fraction of mutations granting resistance almost certainly differs between different antibiotics; for some antibiotics, there will be a greater availability of resistance mutations. Variation in the availability of resistance mutations may reflect differences in the number of genetic loci that can contribute to resistance. For example, resistance to trimethoprim is largely conferred by mutations in a single gene encoding dihydrofolate reductase (DHFR) [10], while resistance to chloramphenicol and streptomycin can be conferred by mutations in a number of different genes [10,11]. Moreover, the number of individual mutations conferring resistance may differ between genes: while a wide range of loss-of-function lesions in the transcriptional regulator *marR* will grant multi-drug resistance [12], only a handful of mutations in *gyrA* confer quinolone resistance [13,14]. Thus, even given equal population size and overall mutation rates, we expect different rates of evolution to resistance to different antibiotics due to differences in the mutational target size.

Once a resistance mutation has arisen, its persistence and spread may be affected by its pleiotropic effects, including its fitness costs and effects on resistance to other drugs [15–18]. In the presence of antibiotic, a resistant bacterium has a clear advantage compared to susceptible genotypes. However, in an antibiotic-free environment, a resistance mutation may impose a burden, for example through reduced growth rates relative to sensitive strains [19–23]. However, while resistance mutations are often costly, not all resistance mutations bear a cost, and such cost-free mutations are likely to persist [24–27]. Melnyk *et al*. (2015) [28] conducted a meta-analysis including 179 single chromosomal mutations conferring resistance to 16 antibiotics from 8 bacterial species. They reported 8 no-cost mutations, with variable costs of resistance depending on antibiotic class and the species assayed.

Increased resistance to one antibiotic may be accompanied by increased cross-resistance to other antibiotics. Cross-resistance is often observed between members of the same class of antibiotic. For example, all quinolones target DNA gyrase and Topoisomerase IV, whose subunits are encoded by the *gyrA/B* and *parC/E* genes, respectively. Resistance to quinolones can be conferred by point mutations affecting specific portions of GyrA and ParC, known as the quinolone resistance-determining regions (QRDR) [13,29–31]. Changes at amino acid positions 83 and 87 of *gyrA* lead to a significant loss in quinolone susceptibility [32–34]. Moreover, known resistance mutations in *gyrB* mutations affect amino acid positions 426 and 464, sites that interact with the bound quinolone molecule close to the QRDR of GyrA [35–37].

Cross-resistance can also occur between drug classes—for example, *marR* mutations selected by quinolones also confer resistance to phenicols, tetracyclines, and rifampicin [31,38,39]. Mutations in *marR*, which encodes a negative regulator of the *marRAB* operon, render the repressor function inactive, resulting in increased efflux and reduced permeability [14,40–43]. By contrast, in collateral sensitivity or negative cross-resistance, acquisition of resistance to one antibiotic may grant sensitivity to other antibiotics. For example, resistance to aminoglycosides in *E*. *coli* can be conferred by electron transport chain mutations that reduce proton-motive force (PMF). This decrease in PMF negatively affects the activity of multi-drug efflux pumps, such as AcrAB-TolC, granting hypersensitivity to many other antibiotics [44,45].

Here, we assess mutation rates, levels of resistance, and pleiotropic effects for chromosomal mutations conferring resistance to different quinolones, in an effort to understand the full set of parameters contributing to the origin, spread, and persistence of quinolone resistance. Quinolones were first used clinically in the 1960’s, and have undergone multiple rounds of development. The first-generation quinolone, nalidixic acid (nal), possesses a limited spectrum of activity, but fluorination of the core structure generated the so-called 2^nd^ -generation quinolones like ciprofloxacin (cip). Further overall structural developments resulted in 3^rd^-generation drugs such as levofloxacin (levo). We predict that, while broad mechanisms of resistance will be shared between quinolones, resistance mutations will differ in their effects towards different antibiotics owing to differences in antibiotic penetration and/or structural configuration.

## Materials and methods

### Bacterial strains and media

The *E*. *coli* laboratory strain K-12 (MG1655) was used for all experiments. Lysogeny broth (LB) (10 g/l tryptone, 5 g/l yeast extract, 10 g/l NaCl per litre; Bishop) was used for agar and broth cultures.

### Quinolone susceptibility assays

Minimum inhibitory concentration (MIC) values for the ancestral strain and for antibiotic-resistant mutants were determined for nalidixic acid, ciprofloxacin and levofloxacin (Sigma-Aldrich) using a 96 well plate assay. Antibiotic concentrations started at 10*μ*g/ml, 1*μ*g/ml, and 8*μ*g/ml for nal, cip, and levo, respectively, and were diluted in a two-fold series and dispensed with 125*μ*l/well of LB into 96-well plates. The 96 well plates were incubated overnight at 30 C, with shaking at 150 rpm. The MIC was defined as the lowest concentration of antibiotic for which 90% growth inhibition was visibly observed after overnight culture.

### Fluctuation analysis and estimation of mutation rates

Mutation rates to resistance were determined using fluctuation assays [46,47]. A single colony of *E*. *coli* MG1655 was grown overnight in liquid LB at 37 C, with shaking at 150 rpm. Fresh 200*μ*l cultures were inoculated with ~100 cells each. Each independent culture was then grown to saturation overnight. The final number of cells, N_t_^,^ was estimated from plate counts on LB without antibiotic. Selective plates were supplemented with antibiotics (nal, cip or levo) at a concentration of 1xMIC or 2xMIC. 30 replicate populations were plated for each antibiotic at each concentration. The observed number of mutants, *r*, was then counted for each replicate.

For estimating the number of mutational events *m*, the MSS maximum-likelihood method was used [48]. This method is based on a recursive algorithm for estimating the Luria-Delbruck distribution for a given number of mutational events [46]. This method is valid over the entire range of values of *m* [49,50]. The mutation rate per cell per generation, *M*, is calculated as *m* divided by the total number of bacteria plated on selective plates (N_t_) [47,51,52].

### PCR amplification and sequencing of candidate genes

Targeted sequencing of the known resistance loci *gyrA* and *marR* was carried out in order to identify potential resistance mutations. To ensure independence, a single mutant colony was picked from every plate from the fluctuation assay on which there was growth, and inoculated overnight in LB broth without antibiotic. DNA was then extracted using the EZ-10 spin column bacterial DNA miniprep kit (Bio Basic) and PCR was used to amplify the QRDR of *gyrA* (Gyrase forward—5’GTAAAACGACGGCCAGTGATGAGCGAC3’, Gyrase reverse—5’CGGTACGGTAAGCTTCTTC3’) and the entire *marR* gene (MarR-forward 5’GTAAAACGACGGCCAGTGGTCAATTCA3’, MarR reverse—5’TCTGGACATCGTCATACCTC3’). PCR amplicons were sent to Genome Quebec for Sanger sequencing. Mutations in these particular regions were compared with the wild type MG1655 strain of *E*. *coli*.

### Whole-genome sequencing

Whole-genome sequencing was carried out to identify potential resistance mutations in clones for which mutations were observed in neither *gyrA* nor *marR*. Sequencing libraries were prepared using the Nextera XT kit (Illumina), and sequencing was carried out on the MiSeq platform using paired-end 300bp reads. Raw reads were processed using Trimmomatic-0.35 [53], allowing for a minimum Phred-scaled quality score of 20 for leading and trailing bases, truncating reads once average quality dropped below 20 in a 4bp sliding window, and dropping all reads of length less than 36. Read quality was assessed using FastQC (https://www.bioinformatics.babraham.ac.uk/projects/fastqc/).

Reference-based assembly was carried out using Bowtie-2 [54], with *E*. *coli* K-12 (MG1655; NC_00913.2) as the reference genome. SNPs were called using Samtools [55] and SNP effects were inferred using SNPeff [56].

### 24-hour growth curve analysis

Fitness in the absence of antibiotic was estimated using 24-hour growth curves for each single colony isolate. Growth curves were obtained in triplicate in 96-well plates, inoculated at a 1:100 dilution from overnight cultures. OD_600_ was measured on a Bioteck ELx808 plate reader every 37 minutes for 24 hours, incubating at 37 C with 30 s of shaking every 5 minutes. Three growth parameters, lag time, maximum growth rate and optical density at stationary phase after 24 hours, were estimated using the program GrowthRates [20].

## Results and discussion

### Estimation of mutation rates

**Fig 1:**
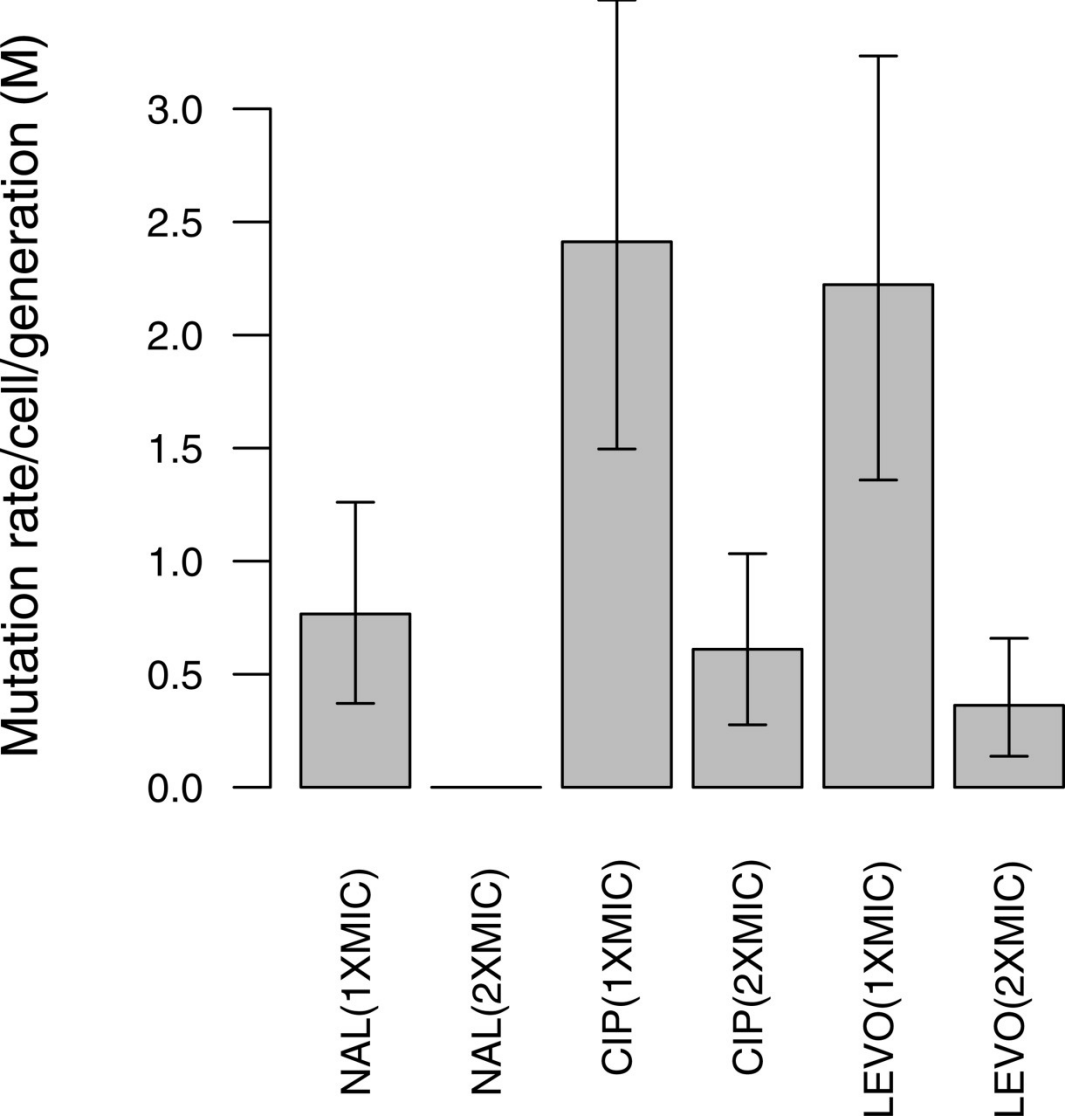
Spontaneous mutation rate per 108 cells to quinolone resistance among *E*. *coli* K-12 (MG1655). Mutation rates were estimated from 30 independent cultures at 1x and 2x MIC. Error bars represent 95% confidence intervals. Note that no colonies were obtained at 2xMIC for Nal.

We estimated mutation rates to resistance for each of the three quinolones nal, cip, and levo using fluctuation assays [46]. The probability of a mutational event per cell per generation was estimated at 1x and 2x MIC where MIC values were 10 μg/ml, 15 ng/ml, and 31 ng/ml for nal, cip, and levo, respectively. The number of observed mutants (r) on each of 30 selective plates was used to estimate mutation rates to nal^R^, cip^R^, and levo^R^, using the MSS maximum likelihood method [50]. At 1x and 2x MIC, mutation rates differed between antibiotics, with lower rates to nal^R^ than to cip^R^ or levo^R^ (cip>levo>nal), presumably because fewer resistance mutations are available for nal that can grant sufficiently high levels of resistance (Fig 1, Table 1).

**Table 1:** Spontaneous mutation rates to quinolone resistance in *E*. *coli* K-12 (MG1655).

Antibiotic	Antibiotic concentration (*μ*g/ml)	Mutation rate per culture ‘m’	Mutation rate (M) per 10^8^ cells	Upper 95% Confidence interval	Lower 95% Confidence interval
Nalidixic acid (1xMIC)	11.11	0.368	0.76	0.49	0.39
Ciprofloxacin (1xMIC)	0.015	1.158	2.41	1.06	0.91
Ciprofloxacin (2xMIC)	0.030	0.293	0.61	0.42	0.33
Levofloxacin (1xMIC)	0.0312	1.067	2.22	1.01	0.86
Levofloxacin (2xMIC)	0.0625	0.174	0.36	0.29	0.22

### Identification of resistance mutations

**Fig 2:**
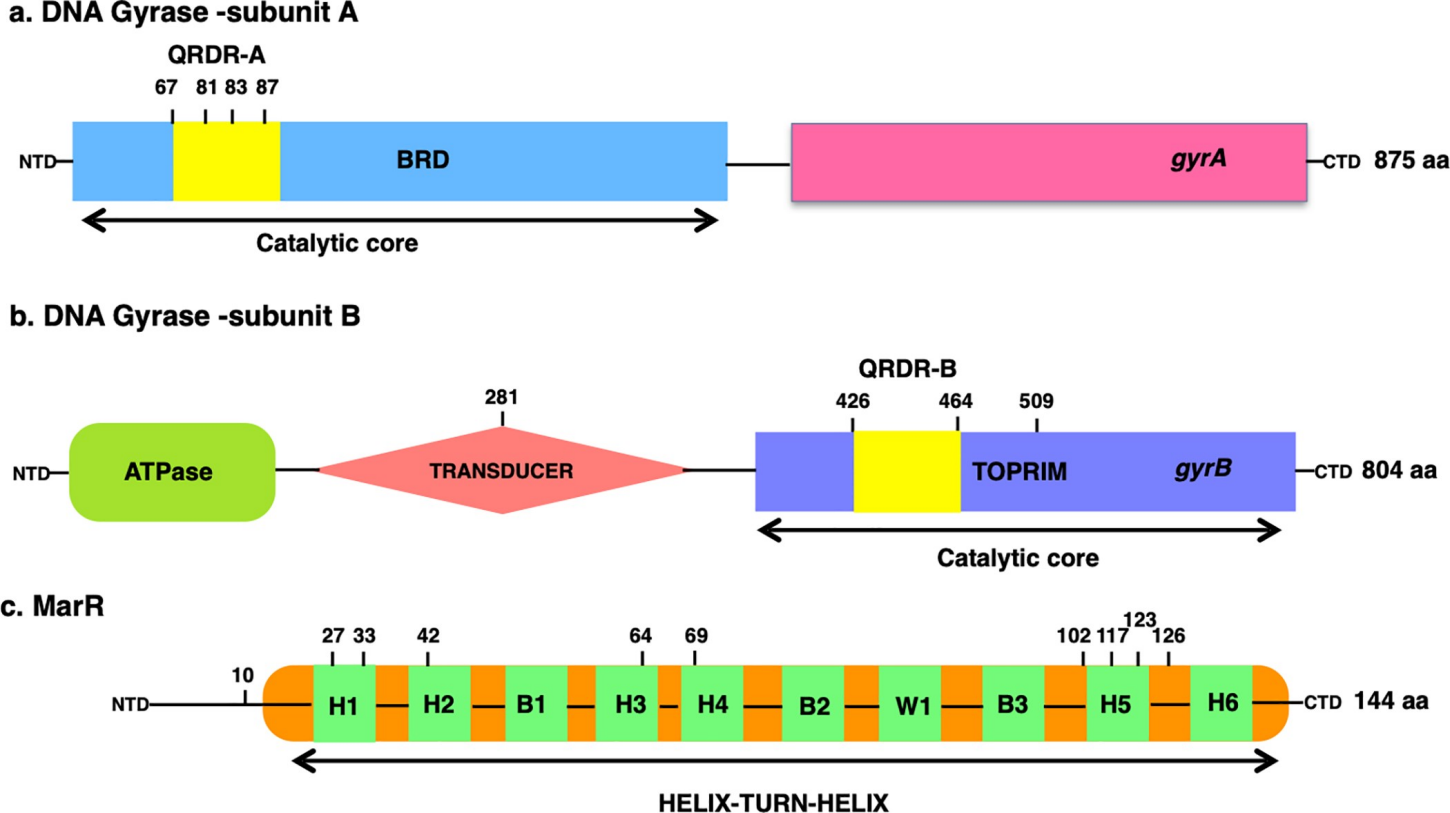
The domain structures of Gyrase A, B, and MarR. Mutations obtained in this study are indicated in bold above. Panel A: Arrangement of GyrA. This subunit of DNA gyrase consists of the breakage-reunion (BRD) domain, and the quinolone resistance determining region (QRDR-A) site. Panel B: Arrangement of GyrB, with the ATPase, Transducer (221–392), and Toprim (418–533) regions. The QRDR-B is shown within the Toprim domain of GyrB [73]. Panel C. MarR domain structure, comprising four helices (H) and three ß-sheets (B). H3 and H4 (57–80) are the recognition and DNA binding motifs containing H-T-H motifs and the ß-sheet winged structure. H1, H5, and H6 are associated with dimerization [42,74–76].

We obtained a total of 56 spontaneous quinolone resistant mutants from the fluctuation assays (one from each plate), and identified putative resistance mutations in 50 of these (Table 2). Targeted sequencing identified 36 *gyrA* mutants, 9 *marR* mutants, and 1 double *gyrA*, *marR* mutant. Whole-genome sequencing identified an additional 4 *gyrB* mutants. We note that *gyrA/gyrB* or *marR/gyrB* double mutants will be undetected by our approach. However, given that only one *gyrA/marR* double mutant was detected, the frequency of double mutants is probably low. In *gyrA*, mutations were observed at nucleotides encoding amino acid positions 67, 81, 83, and 87 (Fig 2A), consistent with previous findings that the GyrA QRDR spans amino acid 67 to 107 [29,57–59]. The most common alterations in the *gyrA* QRDR region were S83L (n = 18) and D87G/N/Y (n = 11). These mutations were found in mutants isolated against all three drugs, presumably because these positions are located within the positively charged region close to the DNA-enzyme binding site [31]. Amino acids 83 and 87 are located near the active site of DNA gyrase, along with the tyrosine-122 residue that interacts with the broken DNA strand following cleavage [59–66]. The α helix-4 region is particularly essential to quinolone binding and the substitution to leucine at position 83 makes the vicinity of α helix-4 of the gyrase less electron rich, crippling gyrase-quinolone binding [33,61,67]. Mutations at position 87 and 81 also perturb the alignment of the α helix-4 structure.

**Table 2:** MIC fold-increase and mutations in *gyrA*, *gyrB* and *marR* among nal^R^, cip^R^, and levo^R^ mutants of *E*. *coli* K-12 (MG1655).

S.No.	Antibiotic	MIC WT (ug/ml)	MIC fold increase of mutants			Resistance mutation			Total number of nal^R^, cip^R^ and levo^R^ mutants	Number of *gyrA*, *gyrB* and *marR* mutants
			Nal	Cip	Levo	*gyrA*	*gyrB*	*marR*		
	Nalidixic acid	10							11	
1			64			A67S				2
2			256			S83L				6
3			128			D87G				2
4			64				D426N			1
	Ciprofloxacin	0.015							24	
1				64		G81C/D				3
2				128		S83L				4
3				32		S83W				2
4				64		D87Y/G/N				7
5				32			H281L			1
6				32			S464Y			1
7				16			L509G			1
	Levofloxacin	0.031							21	
1					8	G81C				1
2					16	S83L				8
4					8	D87G				1
3					16	D87G		R27P		1
5					16			E10stop		1
6					16			L33R		1
7					8			Q42E		1
8					4			L64fs		1
9					16			G69E		1
10					8			T102S		1
11					8			Q117stop		1
12					4			L123S		1
13					8			N126fs		1

*marR* mutations were obtained only in levo^R^ isolates. These point, frameshift, and missense mutations were dispersed throughout the gene, as expected given that the Mar phenotype can arise from any loss-of-function mutation (Fig 2C). MarR consists of two domains, one N-C terminal domain and a helix-turn-helix (HTH) DNA binding domain. The closely packed hydrophobic core and intermolecular hydrogen bonds stabilize the N-terminus (residues 10–21) of one subunit and the C-terminus (residues 123–144) of the second subunit, holding the dimer together. Some of the mutations reported in this study belong to the oligomerization dimer domain of MarR, such as those at positions 10, 27, and 33 of one terminal subunit, and positions 123 and 126 of the other equivalent subunit. The rest of the MarR protein is linked via antiparallel helices emerging out from each of the subunits, encompassing the DNA binding domain (residues 55–100) [12,42]. Mutations at positions 64 and 69 fall in the HTH DNA binding motif. Other mutations reported here at position 42 and 102 residue at α and β sheets of MarR, which are essential for the interaction between the two antiparallel strands of HTH DNA binding domain [42].

WGS revealed *gyrB* mutations in four isolates for which no *gyrA* or *marR* mutations were detected (Fig 2B). H281L, S464F, and L509Q mutations were found in strains isolated on cip, and a D426N mutation was found in a strain isolated on nal. The GyrB enzyme consists of two domains, an N-terminal domain (amino acid 2–393) that incorporates the ATPase catalytic site, and a C-terminal domain (amino acid 394–804) that interacts with GyrA [68,69]. D426N and S464F have been previously reported to confer quinolone resistance [35,59,64,70,71]. Both of these mutations are part of the QRDR of GyrB and cause conformational changes in the structure of the gyrase subunits [37,59]. The *gyrB* D426N mutation has been reported before along with a mutation at position L447, both of which provide a neutral vicinity, owing to their respective opposite charges. Both of these residues are suggested to be part of a quinolone-binding pocket [31,35,36,70,72].

Interestingly, the H281L and L509G mutations have not been previously reported to confer quinolone resistance. These two novel mutations are located outside the GyrB QRDR. Position 281 is located in the transducer region of GyrB, which forms a cavity just large enough to facilitate the transfer of the trapped double-stranded DNA through the DNA gate in the presence of ATP [73,77–79]. Position 509 is within the TOPRIM domain of GyrB, part of the catalytic DNA cleavage-rejoining complex along with the GyrA winged helix domain [79,80].

### Direct responses to selection

**Fig 3:**
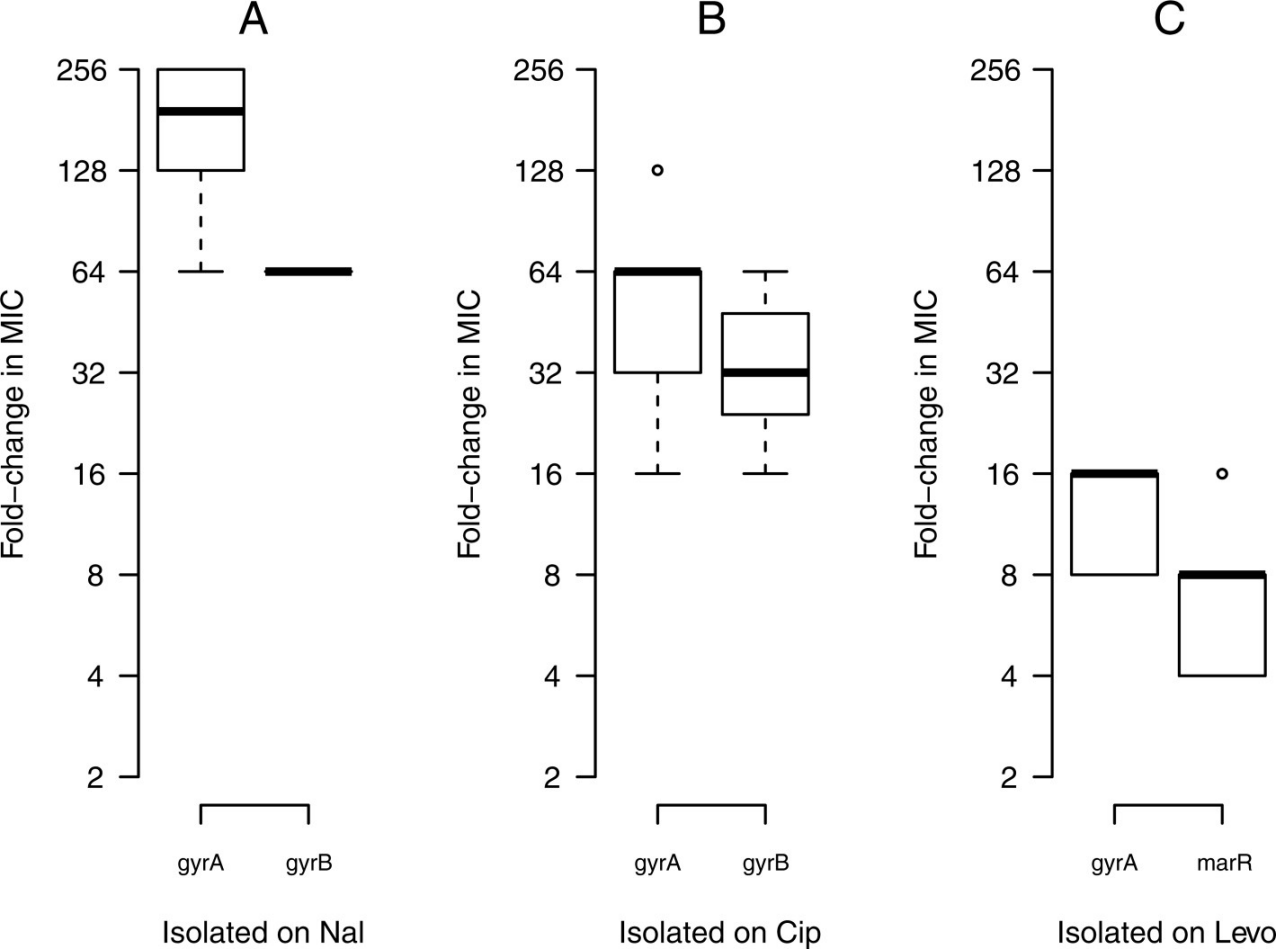
Direct responses to selection. Changes in MIC for resistant mutants towards the drug on which they were selected: nal (A), cip (B), and levo (C). The boxplot presents the median, first, and third quartiles, with whiskers showing either the maximum (minimum) value or 1.5 times the interquartile range of the data, whichever is smaller (larger).

An increase in fitness in the selective environment is referred to as the direct response to selection; here, the direct response to selection is measured by an increase in MIC towards the drug on which a mutant was selected. We found substantial variation between drugs in the magnitude of the direct response. Mutants isolated on nal showed a stronger direct response to selection than did mutants isolated on cip or levo, with a mean increase of 256-fold MIC towards nal. Mutants isolated on cip and levo showed mean increases of 64-fold and 16-fold towards cip and levo, respectively (Fig 3).

Among resistance mutations, *gyrA* mutations consistently showed higher levels of resistance than *gyrB* or *marR*, regardless of the antibiotic they were isolated on, which impacts the variation in MIC values significantly (Table 3). Furthermore, within each gene, the level of resistance varied by mutation. In the case of *gyrA*, the S83L mutation conferred higher resistance among all the isolates compared to other mutational sites of *gyrA* (87, 81 or 67). This suggests that the widespread occurrence of the S83L mutation amongst clinical isolates is due to the high level of resistance (S1 Fig).

**Table 3:** Two-way analysis of variance (ANOVA) for the effects of antibiotic and gene on levels of resistance.

Factor	F-value	*P*-value
Gene	10.00	0.00025*
Antibiotic	39.30	1.34e-10*
Gene*Antibiotic	2.83	0.09

### Cross resistance between quinolones

**Fig 4:**
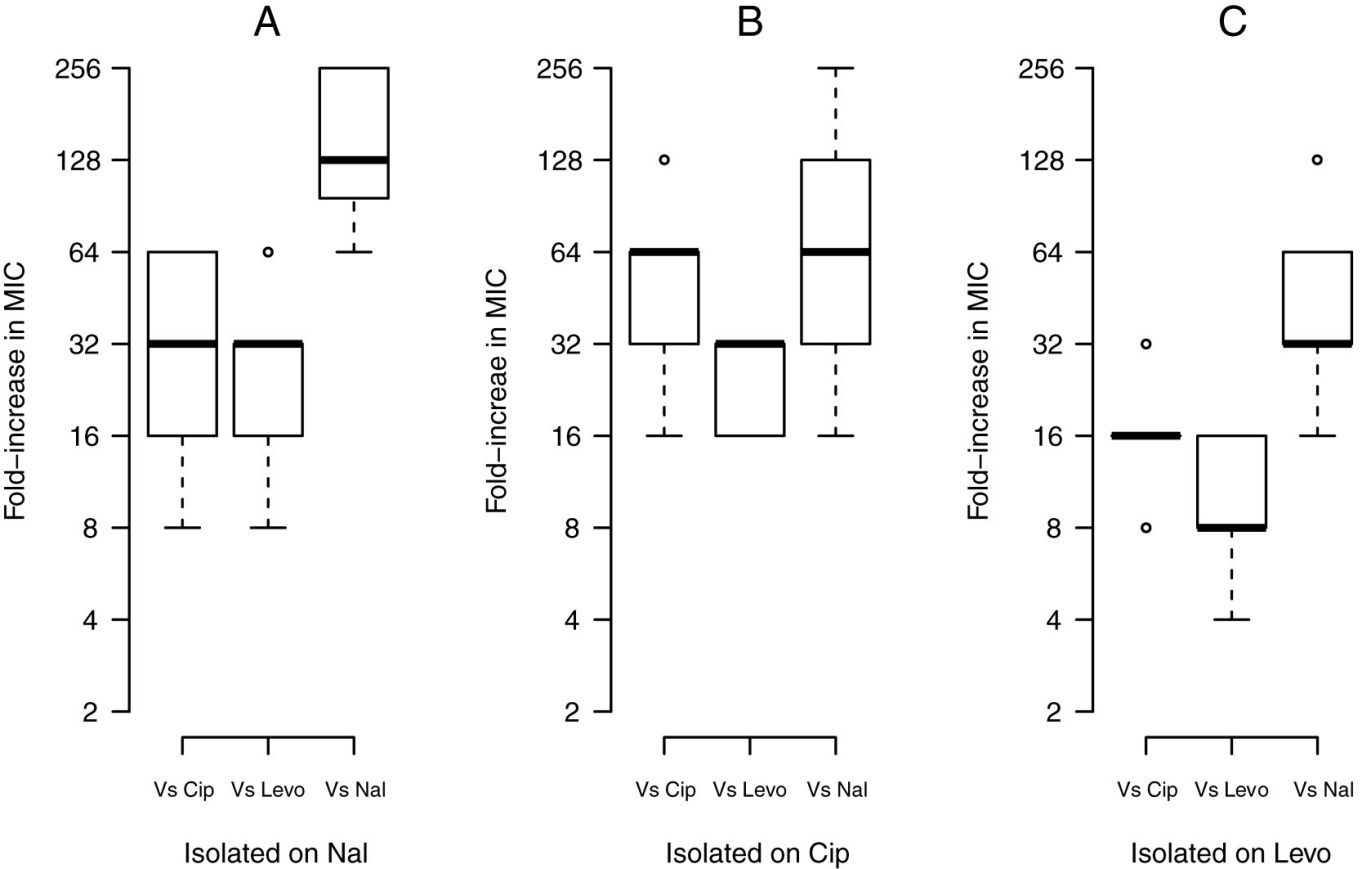
Cross-resistance between antibiotics. Fold-increase in MIC of resistant mutants isolated on nal (A), cip (B), and levo (C) against all three antibiotics. The boxplot presents the median, first, and third quartiles, with whiskers showing either the maximum (minimum) value or 1.5 times the interquartile range of the data, whichever is smaller (larger).

Cross-resistance between quinolones was widespread: all of the resistant mutants isolated on one quinolone displayed increased resistance, in varying degrees, to the other two quinolones. Nonetheless, different quinolones were not equally affected by the resistance mutations (Fig 4). Overall, mutants were more resistant towards nal than they were towards cip or levo. Among cip and levo, mutations showed smaller gains in resistance on levo. Nevertheless, significant correlations between levels of resistance for cip and levo (Pearson’s r = 0.64, t = 6.2, P = 8.433e-08), nal and cip (r = 0.47, t = 3.93, P = 0.0002), and nal and levo (r = 0.38, t = 3.07, P = 0.0032) suggest a closer relationship between the newer quinolones. This variation in resistance among quinolones can be explained by the intrinsic structural drug differences between older and newer quinolone classes. Nal is devoid of any cyclic derivatives whereas cip and levo have substituents at positions C-6, C-7 and C-8, that offer greater spectrum/potency of activity. Thus, the modified quinolone substituents likely reduce resistance levels by increasing the affinity for GyrA, and by stabilizing the quinolone-DNA complex [33,81–96].

These trends are also evident for specific loci. *gyrA* mutants showed the highest gain in resistance (average 128xMIC) on nal in comparison with cip (32xMIC) or levo (16xMIC). *gyrB* mutants also displayed a higher increase in resistance to nal (64xMIC), but the same increase on cip and levo (16xMIC). The novel H281L and L509G mutations gained similar increases in resistance with cip and levo, at 32xMIC and 16xMIC respectively. On nal, H281L gained 64xMIC whereas L509G gained similar increase as with cip or levo, i.e. 16xMIC. Meanwhile, *marR* mutants did not show as high increases in MICs, with increases of 32x, 16x, and 8xMIC on nal, cip, and levo.

### Costs of resistance

**Fig 5:**
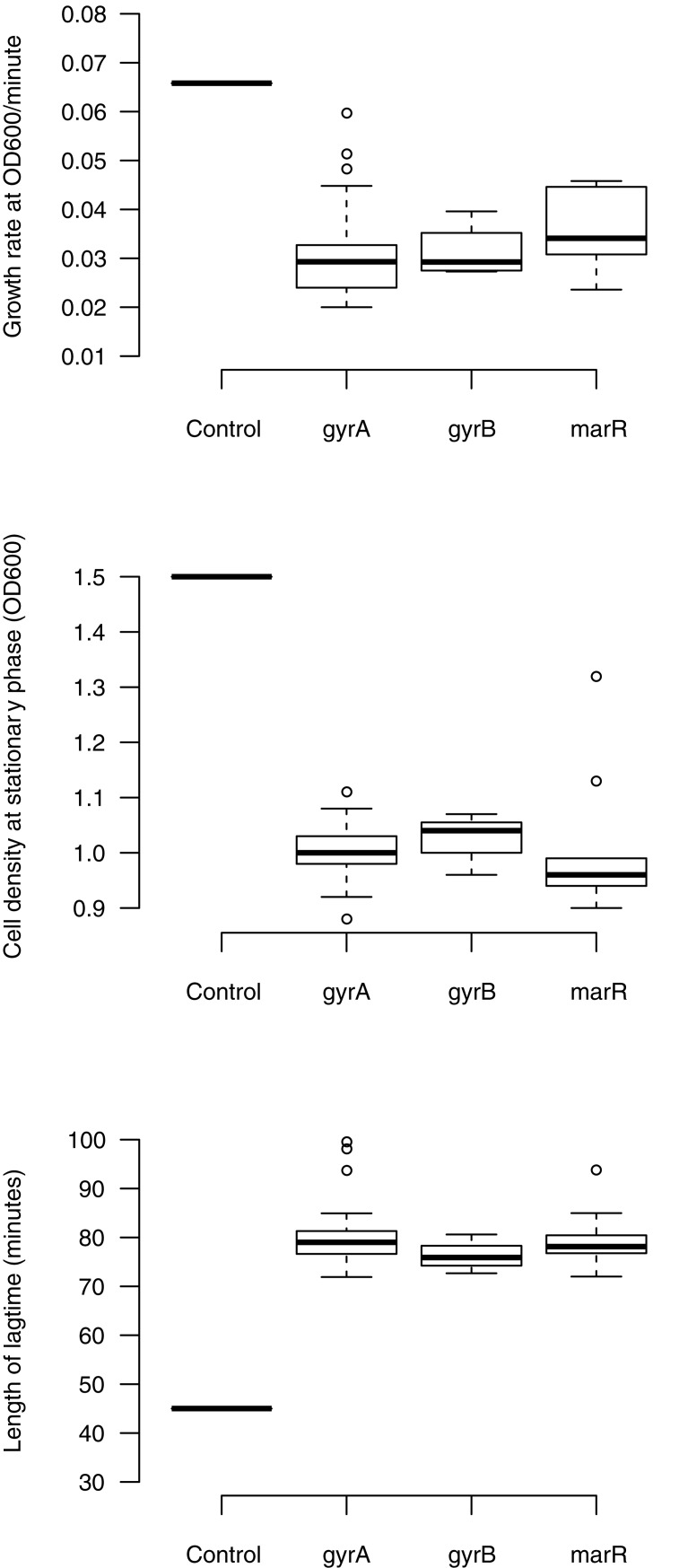
Costs of resistance of quinolone resistant mutants. The fitness components measured are growth rate, cell density, and lag time between *gyrA*, *gyrB*, and *marR* resistance mutations. All the fitness components are compared to control *E*. *coli* K-12 (MG1655). The boxplot presents the median, first, and third quartiles, with whiskers showing either the maximum (minimum) value or 1.5 times the interquartile range of the data, whichever is smaller (larger).

The persistence of resistance in the absence of antibiotic is determined in part by the fitness costs associated with resistance mutations [97–99]. No-cost mutations may contribute to the persistence of resistance mutations in the absence of antibiotic. We measured three fitness components in the absence of antibiotic for our set of quinolone resistant mutants: maximum growth rate (Vmax), density at stationary phase (Max OD), and length of lag phase. Resistant mutants were consistently found to be costly, exhibiting significant differences in Vmax, Max OD, and lag time compared to their drug-susceptible ancestor MG1655 (Fig 5, Table 4) (S2 Fig). Thus, overall we observe significant costs of resistance for quinolone resistance mutations, consistent with previous studies [92,100–102].

**Table 4:** Effects of resistance mutations on growth parameters.

Factor	Post hoc (Tukey HSD) comparisons with *E*. *coli* K-12 MG1655		
	Growth rate Length of Cell density at (OD_600_/minute) lag time (minutes) stationary phase (OD_600_)		
	Mean *P*-value	Mean *P*-value	Mean *P*-value
*gyrA*-MG1655	-0.032 0.0000011	33.4 <2.0e-16	-0.55 <2.0e-16
*gyrB*-MG1655	-0.033 0.0000191	28.9 <2.0e-16	-0.52 <2.0e-16
*marR*-MG1655	-0.029 0.0000173	32.3 <2.0e-16	-0.53 <2.0e-16

**Fig 6:**
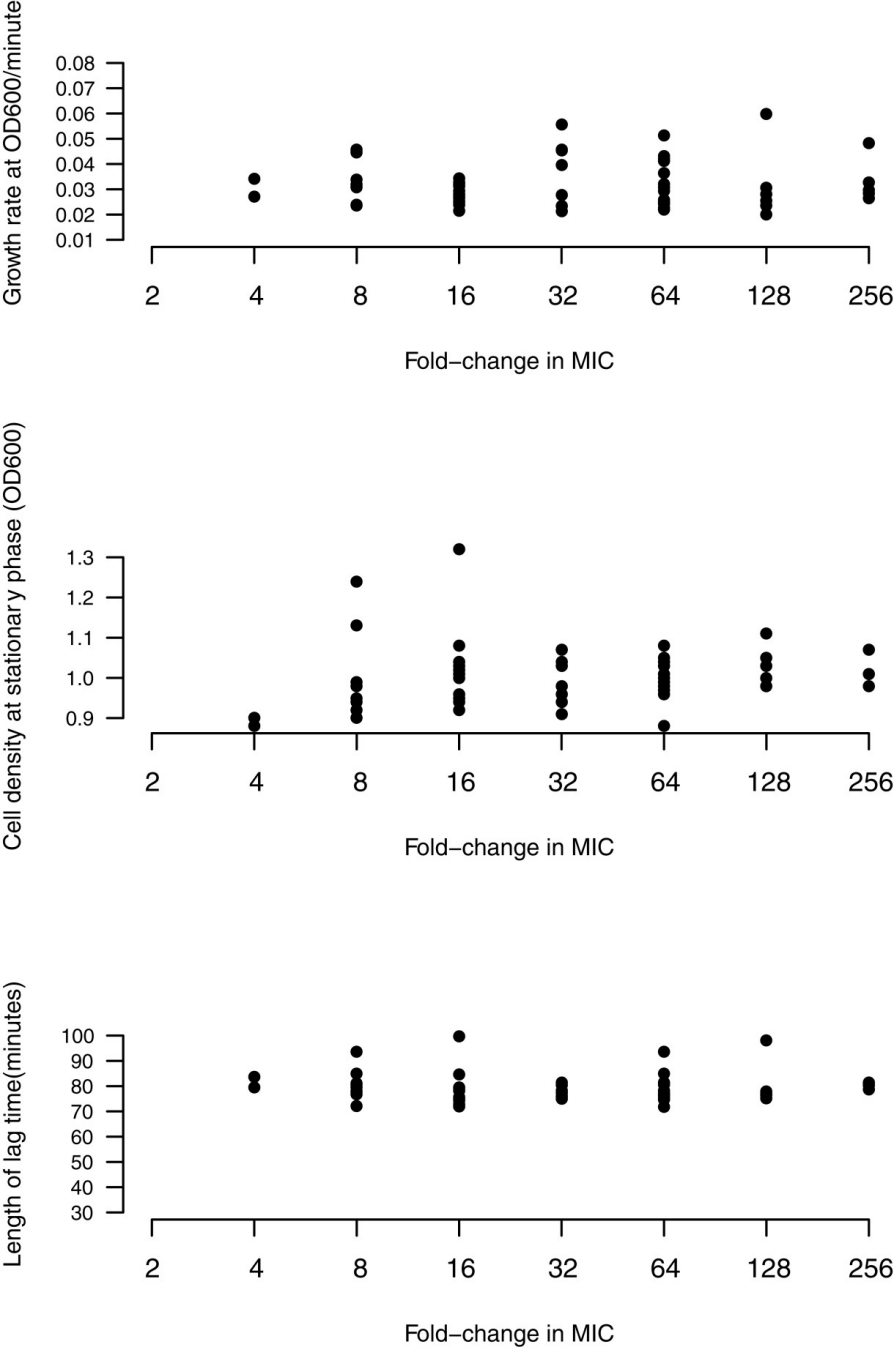
No correlation between level of resistance (fold-increase in MIC) and growth rate, cell density or length of lag phase for all mutants.

Some studies have reported that mutations granting higher levels of resistance impose higher costs [28]. However, we fail to find a significant relationship between MIC and any fitness component. No correlations were found between MIC and growth rate (P = 0.42, Kendall’s tau = 0.08), length of lag phase (P = 0.82, tau = 0.02) or cell density (P = 0.12, tau = 0.15) (Fig 6). We note that *gyrA* mutations confer no greater costs than other resistance mutations (*gyrB*, *marR*). Moreover, amongst the handful of mutations in *gyrA* (S83, D87, G81) that can confer high level resistance [58,63], a few prominent alleles of *gyrA* tend to be found in *E*. *coli* clinical isolates [24,62,103,104]. That these mutations confer high levels of resistance, but are no more costly than other *gyrA* mutations (Fig 6), could help to explain the prevalence of specific *gyrA* mutations amongst clinical isolates.

## Conclusions and perspectives

Quinolones target DNA gyrase and topoisomerase IV. Resistance against these drugs can be achieved by target alteration and/or through efflux and permeability associated mutations. We find that all 50 spontaneous mutants obtained through fluctuation assays were resistant through mutations in the known resistance conferring genes *gyrA*, *gyrB*, and *marR*. This finding suggests that there are few other quinolone resistance mutations available in *E*. *coli* K-12; this is somewhat surprising, given that selection experiments in *P*. *aeruginosa* have identified novel resistance mutations [105]. We find significant costs of resistance, and differences in the mutational supply rate among resistant isolates. Notably, we find *gyrA* mutations conferred higher resistance, without greater fitness cost, than other mutations. This finding may explain the prevalence of *gyrA* mutations in clinical samples. We also find variation in cross-resistance amongst quinolone resistant isolates, implying that different resistance mutations respond differently to quinolone variants. Thus, antibiotic variants may have different implications for the evolution of resistance. Optimally, we should choose an antibiotic for which resistance is costly, and where single mutations have relatively small effect, as was the case here for levofloxacin.

## Supporting information

S1 Fig Comparison between mean fold-increase in MIC values for different resistance mutations in *gyrA*, *gyrB*, and *marR* regions. Resistance mutations isolated as a direct selection on nal (A), cip (B), and levo (C).
(EPS)

S2 Fig **Variation in costs of resistance—Growth rate, cell density, and lag phase between *gyrA* (A), *gyrB* (B), and *marR* (C) resistance mutations.** All the fitness components are compared to control *E*. *coli* K-12 (MG1655). The boxplots present the median, first, and third quartiles, with whiskers showing either the maximum (minimum) value or 1.5 times the interquartile range of the data, whichever is smaller (larger).
(EPS)

S1 File The supplementary data file associated with this article. (XLSX)
